# Epidemiological features of traumatic spinal cord injury in Wuhan, China

**DOI:** 10.1186/s13018-023-03554-6

**Published:** 2023-01-30

**Authors:** Fater A. Khadour, Younes A. Khadour, Ling Meng, Cui Lixin, Tao Xu

**Affiliations:** 1 Department of Rehabilitation, Tongji Hospital, Tongji Medical College, Huazhong University of Science and Technology, 1095#, Jie-Fang Avenue, Qiaokou District, Wuhan, 430030 Hubei China; 2 Department of Rehabilitation, Faculty of Medicine, Al Baath University, Homs, Syria; 3 Physical Therapy Department for Neuromuscular and Neurosurgical Disorder and Its Surgery, Cairo University, Cairo, 11835 Egypt

**Keywords:** Traumatic spinal cord injury, Epidemiology, Prevention, Wuhan

## Abstract

**Background:**

Spinal cord injuries are extremely debilitating and fatal injuries. There is currently little research focusing on traumatic spinal cord injuries, and there is little information available about the epidemiological characteristics of patients with traumatic spinal cord injury (TSCI).

**Objective:**

To describe the epidemiological features of traumatic spinal cord injury in Wuhan, China.

**Design:**

A retrospective hospital-based study.

**Setting:**

Rehabilitation department of Wuhan's Tongji Hospital.

**Participants:**

People who had been diagnosed with a traumatic spinal cord injury (TSCI) were admitted to Tongji Hospital from 2016 to 2021 (*n* = 463).

**Interventions:**

Not applicable. Outcome measures: Epidemiological features such as sex, age, marital status, etiology, occupation, neurological level of injury, and the American Spinal Injury Association Impairment Scale on admission, hospitalization, and concomitant injuries were collected.

**Results:**

The mean age of patients with TSCI was 39.4 ± 14.3 years, and the male/female ratio was 3:1. The leading causes of TSCIs were traffic accidents (38.4%), followed by falls (low falls 24.0%, high falls 13.2%). The most common injury site was the cervical spinal cord, followed by the thoracolumbar level. Of all patients, 463 patients (67.2%) had complications and other injuries. During the hospitalization period, a total of 217 patients experienced complications, with a percentage of 46.9%. Urinary tract infection was the most common (15.6%), followed by pulmonary infection (14.0%).

**Conclusion:**

The results found that the proportion of males was greater, and the first two main reasons were falls and traffic accidents. Farmers and workers are the occupations most vulnerable to SCI. We need to pay more attention to the elderly's risk of falling. These findings suggested that preventive strategies should be based on the features of different types of TSCI patients. Finally, the importance of SCI rehabilitation must be highlighted.

## Introduction

Traumatic spinal cord injury (TSCI) is one of the most devastating and debilitating conditions that can significantly impair sensory, motor abilities, and autonomic function [1]. It is regarded as the second leading cause of morbidity and disability after traumatic brain injury [2]. TSCI has devastating psychological, physical, and economic implications [3]. Additionally, TSCI places a heavy burden on society and individuals with SCI, including a negative effect on the quality of life (QoL) and considerable economic burdens for healthcare services [4].

Although the prevalence of spinal cord injury has increased in tandem with economic development, there has not seemed to be significant progress in clinical care, and proactive prevention appears to become the best strategy [5].

According to a global epidemiological survey done in 2010, the prevalence of TSCI ranged from 13.1 to 52.2 per million individuals [6]. The estimated annual incidence of TSCI varies considerably depending on each country. It ranges from 10.6 per million in North America to 84 per million in Western Europe [3, 5]. Whereas, the estimated amount of TSCI in Asia ranged from 12.06 to 61.6 per million in 2012 [7]. This is correlated with the various levels of economic development in each country.

In China, the recent annual incidence was 60.6 per million population in Beijing and 23.7 per million in Tianjin [8, 9]. China, unlike the majority of developed countries, appears to lack a national spinal cord injury registration system [6]. Because no nationwide database of SCIs has been established in mainland China, extant research in the literature has all been hospital-based [10], with the majority of these studies concentrated on TSCIs [11, 12]. There have also been some hospital-based studies that have concentrated on epidemiological and injury features rather than the incidence rate of TSCI [11, 13]. Because each geographic location has its own epidemiological features, performing such epidemiological studies at the current population level is essential.

Wuhan is considered one of the largest cities in Hubei Province. It is located in central China, with an area of 8494 km^2^ and an estimated 8.5 million residents as of 2022.

To our knowledge, there is little available and documented about the epidemiological features of TSCI in Wuhan. This study aimed to present epidemiologic data on people with TSCI who were treated at Tongji Hospital in Wuhan city from January 1, 2016, to December 31, 2021. And to assist in determining the appropriate use of healthcare resources to reduce social and financial difficulties.

## Methods

To the best of our knowledge, no national population-based TSCI registration system has been established in Wuhan.

Medical records from the rehabilitation department of Tongji Hospital were reviewed retrospectively between 2016 and 2021, using the International Classification of Disease Version 10 (ICD-10) and the TSCI diagnosis code.

This study included the following data; age, sex, etiology of injury, occupation, American Spinal Injury Association (ASIA) Impairment Scale (AIS), level of injury, and accompanying injuries.

Inclusion criteria for the study were spinal cord injuries or cauda equina injuries induced by trauma in Wuhan and patients hospitalized at Tongji Hospital at the time of injury. And the International Spinal Cord Injury Core Data Set (version 1.1) was employed for this study.

The study's exclusion criteria included intervertebral disk disease, spinal fractures without SCI, patients with incomplete medical files, medical files with unclear diagnoses, and fatally injured individuals who were never admitted to the hospital.

In the current study, the participants were divided into six age groups like in many previous studies [14]. 0–19, 20–29, 30–39, 40–49, 50–59, 60 and above. Marital status was recorded as (married, unmarried, divorced, and widowed).

The etiology of injury was classified as a traffic accident (Four-wheeled vehicles, Two-wheeled vehicles, Bicycles, and Pedestrians) and falls (for low falls, height < 1 m; for high falls, height ≥ 1 m) [15]. Injuries caused by falling objects, machinery-related injuries, and sports-related injuries.

Occupations included workers, farmers, government- offices, students, retired, and others. The neurological level of injury comprised cervical, thoracic, lumbar, and sacral segments.

### Ethical considerations

This study was approved by the ethics committee of Tongji Hospital, Tongji Medical College, TJ-IRP20220314.

### Data analysis

The statistical analyses were performed with the assistance of version 23.0 of the SPSS for Windows software package. The data were then arranged on an Excel spreadsheet for statistical and tabulation purposes. Descriptive statistics were computed describing the baseline variables. Characteristics of the patients were presented as mean value ± standard deviations. Frequency analysis was employed to analyze data and calculate percentages. The analysis of variance Chi-squared and (ANOVA) tests were used to analyze categorical a continuous data, respectively. The level of statistical significance was preset at *p* < 0.05.

## Results

From 2016 to 2021, 507 TSCI cases were detected, and 44 were excluded from this research study (intervertebral disk disease 9; spinal column fracture without SCI 16; incomplete medical records or uncertain diagnosis 19.

### General characteristics of TSCI patients between 2016 and 2021

The demographic features of TSCI people are shown in Table 1. Of the 463 people with TSCI, 349 (75.4%) were male, and 114 (24.6%) were female. Thus, the male-to-female ratio was 3:1.

**Table 1:** Characteristics of patients with TSCI from 2016 to 2021

Years	2016	2017	2018	2019	2020	2021	Total
*Age*							
0–19	0 (0.00%)	3 (0.64%)	5 (1.07%)	5 (1.07)	6 (1.29%)	5 (1.07%)	24 (5.18%)
20–29	5 (1.07%)	6 (1.29%)	7 (1.51%)	19 (4.10)	23 (4.96%)	21 (4.53%)	81 (17.49%)
30–39	23 (4.96%)	27 (5.83%)	21 (4.53%)	35 (7.55%)	34 (7.34%)	42 (9.07%)	182 (39.30%)
40–49	15 (3.23%)	13 (2.80%)	10 (2.15%)	16 (3.45%)	13 (2.80%)	16 (3.45%)	83 (17.92%)
50–59	8 (1.72%)	10 (2.15%)	8 (1.72%)	6 (1.29%)	10 (2.15%)	6 (1.29%)	48 (10.36%)
≥ 60	8 (1.72%)	11 (2,73%)	4 (0.86%)	6 (1.29%)	7 (1.51%)	9 (1.94%)	45 (9.71%)
*Gender*							
Male	49 (10.58%)	54 (11.66%)	41 (8.85%)	67 (14.47%)	68 (14.68)	70 (12.43%)	349 (75.37%)
Female	10 (2.15%)	16 (4.45%)	14 (3.02%)	20 (4.31%)	25 (5.39%)	29 (6.26%)	114 (24.62%)
*Education level*							
Illiterate	6 (1.29%)	3 (0.64%)	1 (0.21%)	0 (0.00%)	1 (0.21%)	1 (0.21%)	12 (2.59%)
Elementary school	23 (5.0%)	17 (3.76%)	10 (2.15%)	10 (2.15%)	18 (3.88%)	11 (2.37%)	89 (19.22%)
Middle school	16 (3.45%)	27 (5.83%)	19 (4.10%)	37 (7.99%)	34 (7.34%)	45 (9.71%)	178 (38.44%)
High school	4 (0.86%)	11 (2.37%)	12 (2.59%)	18 (3.88%)	21 (4.53%)	24 (5.18%)	90 (19.43%)
College or more	10 (2.15%)	12 (2.59%)	13 (2.80%)	22 (4.75%)	19 (4.10%)	18 (3.88%)	94 (20.30%)
*Occupation*							
Farmer	11 (2.37%)	8 (1.72%)	9 (1.94%)	18 (3.88%)	13 (2.80%)	17 (3.67%)	76 (16.38%)
Worker	22 (4.75%)	19 (4.10%)	14 (2.99%)	24 (5.18%)	28 (6.04%)	32 (6.91%)	139 (30.02%)
Government- offices	6 (1.29%)	10 (2.15%)	8 (1.72%)	14 (2.48%)	14 (3.02%)	16 (3.45%)	68 (14.68%)
Retired	5 (1.07%)	6 (1.29%)	4 (0.86%)	4 (0.86%)	5 (1.07%)	5 (1.07%)	29 (6.26%)
Students	2 (0.43%)	2 (0.43%)	5 (1.07%)	10 (2.15%)	11 (2.37%)	8 (1.72%)	38 (8.20%)
Others*	13 (2.80%)	25 (5.39%)	15 (3.23%)	17 (3.67%)	22 (4.75%)	21 (4.53%)	113 (24.40%)
*Etiology*							
Traffic accidents	22 (4.75%)	24 (5.18%)	24 (4.18%)	41 (8.85%)	42 (9.07%)	47 (10.15%)	178 (38.44%)
Low fall	10 (2.15%)	9 (1.94%)	12 (2.59%)	13 (2.80%)	24 (5.18%)	21 (4.53%)	111 (23.97%)
High fall	12 (2.59%)	16 (3.45%)	4 (0.86%)	12 (2.59%)	7 (1.21%)	10 (2.15%)	61 (13.17%)
Falling objects	7 (1.51%)	8 (1.72%)	7 (1.51%)	9 (1.94%)	4 (0.86%)	3 (0.64%)	38 (8.20%)
Machinery-injury	6 (1.29%)	10 (2.15%)	5 (1.07%)	6 (1.29%)	12 (2.59%)	13 (2.80%)	52 (11.23%)
Sport	2 (0.43%)	3 (0.64%)	3 (0.64%)	6 (1.29%)	4 (0.86%)	5 (1.07%)	23 (4.96%)
*Marital status*							
Married	41 (8.85%)	51 (11.01%)	31 (6.69%)	56 (12.09%)	62 (13.39%)	73 (15.76%)	314 (67.81%)
Unmarried	13 (2.80%)	9 (1.94%)	15 (3.23%)	21 (4.53%)	21 (4.53%)	19 (4.10%)	98 (21.16%)
Divorced	1 (0.21%)	8 (1.72%)	6 (1.29%)	8 (1.72%)	6 (1.29%)	4 (0.86%)	33 (7.12%)
Widowed	4 (0.86%)	2 (0.43%)	3 (0.64%)	2 (0.43%)	4 (0.86%)	3 (0.64%)	18 (3.88%)
*Ethic groups*							
Han	54 (11.66%)	62 (13.39%)	44 (9.50%)	82 (17.71%)	89 (19.22%)	86 (18/57%)	417 (90.05%)
Zhuang	1 (0.21%)	4 (0.86%)	4 (0.86%)	2 (0.43%)	1 (0.21%)	4 (0.86%)	16 (3.43%)
Miao	2 (0.43%)	3 (0.64%)	4 (0.86%)	2 (0.43%)	1 (0.21%)	4 (0.86%)	16 (3.43%)
Tujia	2 (0.43%)	1 (0.21%)	3 (0.64%)	1 (0.21%)	2 (0.43%)	5 (1.07%)	14 (2.99%)

Other* included unemployed individuals and self-employed individuals

The age of the patients ranged from 11 to 86 years, with a mean age of 39.4 ± 14.3 years. Furthermore, the 30–39-year age group had the largest (39.3%), followed by 40–49 years (17.9%).

Concerning educational level, more than half of the participants finished elementary or middle school (57.7%; *n* = 267), while (2.6%; *n* = 12) of the participants were illiterates.

The most frequent occupational was a worker (30.0%; *n* = 139), followed by other (unemployed individuals and self-employed individuals) (24.4%; *n* = 113) and farmer (16.4%; *n* = 76) (Table 1).

Most patients, (67.8%; *n* = 314) were married and 21.2% (98) were unmarried (Table 1). The dominant ethnic group was Han, which made up 90.1% of the total population, and other ethnic groups were Miao and Zhuang (3.4%), and Tujia (3.0%) (Table 1).

### Etiology of injury

The etiology of TSCI is summarized in Table 1. The results demonstrated that the primary cause of SCI was traffic accidents (38.4%; *n* = 178), followed by falls (low falls 24.0%: *n* = 111, high falls 13.2%: *n* = 61), and machine-related injury (11.2%; *n* = 52). In addition, several unusual causes, including fall objects (8.2%; *n* = 38), and sport (5.0%; *n* = 23), were identified.

**Fig. 1:**
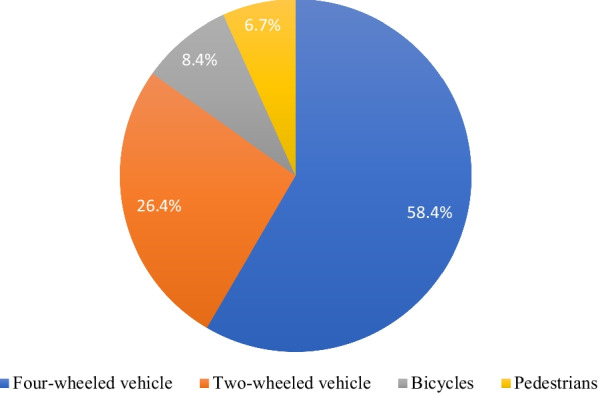
Details of traffic accidents

This study shows that the most frequent cause of traffic accidents was four-wheeled vehicles (58.4%), followed by two-wheeled vehicles (26.4%) (Fig. 1). Traffic accidents were the primary cause of injury for male patients (32.6%; *n* = 151), whereas low falls were the leading cause of injuries for female patients (10.6%; *n* = 49) Table 2.

**Table 2:** Analysis of the etiologies and gender among patients with TSCI from 2016 to 2021

Etiologies	Gender	
	Male	Female
Traffic accident	151 (32.61%)	27 (5.83%)
Low fall	62 (13.39%)	49 (10.58%)
High fall	49 (10.58%)	12 (2.59%)
Falling objects	32 (6.91%)	6 (0.12%)
Machinery-injury	41 (8.85%)	11 (2.37%)
Sport	14 (3.02%)	9 (1.49%)

Table 3 shows the etiologies of injuries in various age groups. Traffic accidents, falls (low and high falls), and machine-related injury were the most frequent etiologies in the 30–39 age group. In addition, most of the injury occurred at the 30–39 age group (39.3%; *n* = 182), followed by the 40–49 age group (17.7%; *n* = 82).

**Table 3:** Analysis of the etiologies and age distribution among patients with TSCI from 2016 to 2021

Etiologies	Age					
	0–19	20–29	30–39	40–49	50–59	≥ 60
Traffic accident	10 (2.15%)	25 (5.39%)	67 (14.47%)	29 (6.26%)	25 (5.39%)	22 (4.75%)
Low fall	4 (0.86%)	22 (4.75%)	33 (7.12%)	25 (5.39%)	9 (1.94%)	18 (3.88%)
High fall	2 (0.43%)	11 (2.37%)	28 (6.04%)	14 (3.23%)	4 (0.86%)	1 (0.21%)
Falling objects	1 (0.21%)	5 (1.07%)	22 (4.75%)	4 (0.86%)	3 (0.64%)	3 (0.64%)
Machinery-injury	1 (0.21%)	14 (3.02%)	22 (4.75%)	10 (2.15%)	4 (0.86%)	1 (0.21%)
Sport	6 (1.29%)	4 (0.86%)	10 (2.15%)	0 (0)	3 (0.64%)	0 (0)

### Level of injury

**Fig. 2:**
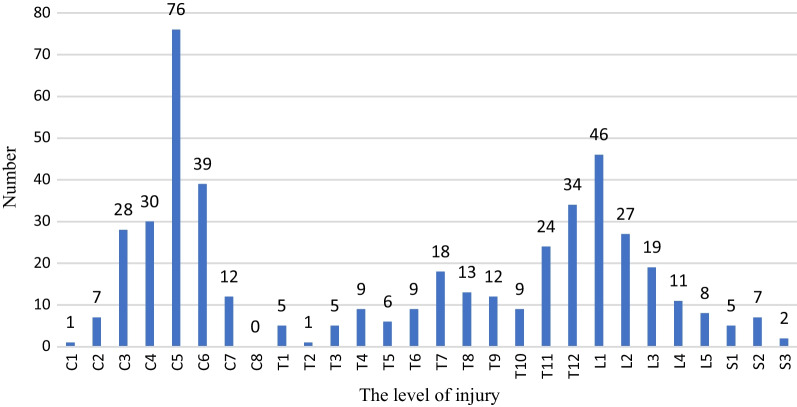
Distribution histogram of the injury level of the patients

This study found a bimodal distribution of TSCI levels, as illustrated in Fig. 2. Injury to the cervical region formed the first peak, while thoracolumbar area injury comprises the second peak.

Based on the ASIA impairment scale, the frequency of grade A, B, C, and D injuries was 157 (33.9%), 165 (35.6%), 100 (21.6%), and 41 (8.9%), respectively.

The different level of injury was associated with different grades of injury. Most people with a cervical level injury 74 (16.0%) were categorized as Grade A, followed by Grade B 67 (14.5%).

While people with thoracic region injury represented the majority 56 (12.09%) of the Grade B (Table 4).

**Table 4:** Comparison of the degrees and segments of the injury among patients with TSCI

ASIA scale	The level of injury			
	Cervical cord (%)	Thoracic cord (%)	Lumbar cord (%)	Sacral cord (%)
A	74 (15.98%)	48 (10.36%)	34 (7.34%)	1 (0.21%)
B	67 (14.47%)	56 (12.09%)	37 (7.99%)	5 (1.07%)
C	35 (7.55%)	26 (5.61%)	21 (4.53%)	18 (3.88%)
D	15 (3.23%)	16 (3.45%)	7 (1.51%)	3 (0.64%)

Furthermore, the different severities of SCI injury were associated with various causes: traffic accident and low falls, grade B injuries, and high falls, grade A injuries (Table 5).

**Table 5:** Analysis of the causes, segments, and degrees of injury among patients with TSCI

Etiologies	ASIA scale			
	A (%)	B (%)	C (%)	D (%)
Traffic accident	57 (11.66%)	62 (13.39%)	47 (10.15%)	12 (2.59%)
Low fall	33 (7.12%)	38 (8.20%)	20 (4.31%)	20 (4.31%)
High fall	29 (6.26%)	24 (5.18%)	5 (1.07%)	3 (0.64%)
Falling objects	10 (2.15%)	17 (3.67%)	8 (1.72%)	3 (0.64%)
Machinery-injury	22 (4.75%)	17 (3.67%)	11 (2.37%)	2 (0.43%)
Sport	6 (1.29%)	1 (0.21%)	9 (1.94%)	1 (0.21)

Etiologies	The level of injury			
	Cervical cord (%)	Thoracic cord (%)	Lumbar cord (%)	Sacral cord (%)
Traffic accident	81 (17.49%)	55 (11.78%)	30 (6.47%)	12 (2.59%)
Low fall	44 (9.50%)	31 (6.69%)	28 (6.04%)	8 (1.72%)
High fall	35 (7.55%)	21 (4.53%)	5 (1.07%)	0 (0.00%)
Falling objects	10 (2.15%)	15 (3.23%)	12 (2,59%)	1 (2.15%)
Machinery-injury	14 (3.02%)	19 (4.10%)	16 (3.45%)	3 (0.64%)
Sport	7 (1.51%)	5 (1.07%)	8 (1.72%)	3 (0.64%)

In addition, the different neurological levels of SCI were associated with various causes: traffic accidents and falls (for low falls, height < 1 m; for high falls, height ≥ 1 m), cervical level, and falling objects, machinery-related injuries, thoracic level (Table 5).

### Treatment of TSCI and clinical complications disturbances of function among SCI individuals

Of all patients, 54.2% (251 /463) underwent surgical procedures like laminoplasty, spinal decompression, fusion, and internal fixation, whereas the proportions of patients accepting rehabilitation therapy, traditional therapy, hyperbaric oxygen therapy were 60.9% (282/463), 53.8% (249/463), and 26.4% (122/463), respectively (Table 6).

**Table 6:** The treatment options for persons with TSCI

Treatment options	Numbers (%)
*Surgery*	
Yes	251 (54.21%)
No	212 (45.78%)
*Rehabilitation therapy*	
Yes	282 (60.90%)
No	181 (39.09%)
*Traditional therapy*	
Yes	249 (53.77%)
No	214 (46.22%)
*Hyperbaric oxygen therapy*	
Yes	122 (26.43%)
No	341 (73.65%)
*Assistive devices*	
Yes	234 (41.56%)
No	229 (49.46%)
*Medicine*	
Yes	75 (16.19%)
No	388 (83.80%)

The hospitalization time for individuals with TSCI varied from 3 to 289 days, with an average of 20.3 days and a standard deviation of 55.7.

A detailed analysis of medical data found that the most frequent related injuries were limb and rib fractures 17.9% (83/463) 16.0% (74/463), pelvic fractures 13.8% (64 /463), craniocerebral injury 10.6% (49 /463), and other injuries, such as lung contusion, hemopneumothorax 8.9% (41 /463).

In the current study, 46.9% (217/463) of the patients with TSCI experienced clinical complications. Urinary tract infection was the most common 15.6% (72/463), followed by pulmonary infection 14.0% (65/463), bedsores 8.0% (37/463), hyponatremia 5.2% (24/463), deep venous thrombosis 2.4% (11/463), and others 1.7% (8/463).

## Discussion

A recent systematic analysis of 17 research in China found that the epidemiological characteristics of SCI varied by region; thus, customized preventative measures should be performed by region.

This is a retrospective cross-sectional research of TSCI patients' features in Wuhan, China, from 2016 to 2021. As a retrospective study, it was unavoidable that some data would be lost. Data loss was reduced by reviewing all associated medical records to get a data set that was as complete as possible.

The current findings showed that the male-to-female ratio of TSCI patients was roughly 3.0:1, which is consistent with the findings of a study conducted in Guangdong [16].

Furthermore, the data revealed that the average age of patients with TSCI was (39.4 ± 14.3) years, and that the majority of injuries occurred in the 30–39-year-old age group, followed by the 40–49-year-old age group. These findings are in line with a recent study conducted in Northwest China, which indicated that most patients were between the ages of 30 and 39 [14].

These findings could be attributed to the fact that the majority of the women work as housewives or in activities with minimal low risk, but males are more likely to be employed in dangerous occupations, especially adult men who conduct more hazardous outdoor work.

These findings are in line with recent studies from other nations [17–20], where people in this age group are active and socially productive members of society.

In traditional Chinese culture, young and middle-aged people are responsible for supporting and raising their parents' families. Additionally, as China's elderly population rises, more elders are experiencing SCIs. These patients may have concomitant conditions, including osteoporotic compression fractures or degenerative spine disease which make them more susceptible to SCI injury [14].

The most prevalent occupational groups in this study were workers (30.0%) and peasants (16.4%). This outcome is consistent with previous results from Tianjing and Chongqing [13, 15].

This finding was primarily due to these patients' low educational background, which restricted them to manual labor and raised their susceptibility to TSCI, which may explain the higher burden of TSCI in this occupational category.

Our findings showed that the proportion of married patients is higher than that of unmarried patients, which could be because most patients were middle-aged, when most Chinese people marry.

Furthermore, this study found that the primary causes of TSCI in Wuhan were traffic accidents (38.4%), falls (low and high falls) (37.2%), machine-related injury (11.2%), falling objects (8.2%), and sport-related injury (5.0%). These findings contradict previous studies, which revealed that falls (52.3%) were the primary cause, followed by motor vehicle accidents (36.4%) [8, 13, 15].

Traditionally, traffic accidents have been the main cause of SCI in most developed countries, and the majority of high falls resulting in TSCI happened in the construction industry [21–23].

This can be attributed to China's quickening industrialization process and its growing massive infrastructure projects. Therefore, it is necessary to significantly develop fall prevention measures in the construction industry.

High-energy injuries, such as those caused by traffic accidents and falling objects, were the leading causes of TSCI in young individuals. In contrast, low-energy injuries, such as those caused by low falls, were more common in the elderly [24]. SCIs are caused by various factors, including falls (both high and low), traffic accidents, impact with falling objects, sports, and violent injuries, and these factors differ by country and region.

In 2006, an epidemiological survey in Canada revealed that traffic accidents were the leading cause of SCIs, while falls (both high and low) became the leading cause in 2009 [25, 26].

Another survey study from seven Middle Eastern and North African (MENA) countries showed that traffic accidents are still the primary cause of SCIs, followed by falls (both high and low), violence, and sports [27].

As this study showed that four-wheeled vehicles were the leading cause of traffic accidents (58.4%), followed by two-wheeled vehicles (26.4%). This outcome is consistent with the survey study conducted in Japan among TSCI patients, which showed that four-wheeled vehicles were responsible for (46.3%) of traffic accidents, while two-wheeled vehicles were responsible for (26.6%) [28].

Cervical injuries were the most frequent level of injury identified in the current study, accounting for 41.3% of all cases. Previous research also showed that 55% to 75% of all spinal cord injuries were cervical injuries, which were the most frequent level [26, 27].

These findings could be explained by the cervical vertebrae's comparatively low mechanical stability, which makes them more prone to trauma than any other part of the vertebral column.

During this period, the percentages of ASIA A, B, C, and D injuries were 33.9%, 35.6%, 21.6%, and 8.9%, respectively, and ASIA A and B injuries comprised the majority of TSCI cases.

The analysis of injury locations in this study revealed a bimodal distribution, similar to other studies' findings, with cervical region formed the first peak, while thoracolumbar area injury comprises the second peak [15, 29].

While injuries from traffic accidents and falls from great heights primarily result in complete SCIs, typically of grade A, falls from a low height mostly result in incomplete SCI, mainly grade B SCIs. Patients with grade A SCIs are more prone to develop depressive disorders and suicide, according to Williams et al. [30] and Thietje et al. [31]; consequently, family and clinicians should provide additional care to these patients to avoid suicide induced by depression.

Although incomplete injuries were more common than complete injuries, first aid technologies should be developed so that people with SCI receive timely and appropriate treatment.

In addition, we revealed an association between the level of injury and the ASIA grade. Where most cervical injuries were graded A, whereas the thoracic injuries were mostly graded B.

It is well known that patients with TSCI experience many complications. Based on the current findings, 46.9% of TSCI patients developed clinical complications, and the four most frequent complications were urinary tract infections (15.6%), pulmonary infections (14.0%), bedsores (8.0%), and hyponatremia (5.2%). These findings are congruent with a previous study performed in northwest China. That study revealed that (36.4%) of people with TSCI experienced one or more complications, and the most frequent complications were pulmonary infections (32.5%), followed by hyponatremia (24.1%), bedsores (16.3%), and urinary tract infections (12.5%) [14].

In addition, another study conducted in Italy showed that the four most frequent complications of TSCI were pain, urinary tract infections, lung infections, and bedsores [32].

Urinary tract infections and pulmonary infections were considered the most common complications among people with TSCI, and they were primarily attributed to inadequate nursing practices in hospitals [32].

Respiratory problems are associated with long-term bed rest, rib fractures, and smoking-related respiratory disease. In addition, SCIs at the cervical level can impair diaphragm or intercostal muscle function, impede respiration, and rise to cough, making sputum removal difficult. Such symptoms may also appear as consequences of respiratory disease [33].

The risk of pulmonary infection increases with a high level of SCI injury. SCI above the C5 level leads to dysfunction of the diaphragm and the risk of a pulmonary infection can increase up to 90% [34]. These findings highlight the need to provide adequate care to patients to reduce hospitalization time and improve prognosis.

## Limitations

This study has several limitations that have to be considered. First, the current study was a hospital-based descriptive study that determined only a small proportion of all people with TSCI; furthermore, Hubei Province does not currently have an SCI-registered system; thus, the exact incidence rate could not be established. Second, the data are collected retrospectively. Consequently, some data loss was unavoidable. Third, we missed some data since many complications and treatments were not adequately diagnosed or documented in medical records. Fourth, people who died at the scene of the accident or on the way to the hospital were not included, which may have resulted in an underestimating of the prevalence rate.

## Conclusion

The evaluation and analysis of the epidemiological features of SCI in Hubei province, China, indicate the need for additional research on the epidemiology of SCI in this province. Furthermore, preventive efforts should be focused on individuals most vulnerable to injury, such as young male adults engaged in dangerous outdoor jobs, and environmental adjustments should be reinforced. Additionally, roads should be expanded and protective barriers placed between the motorway and the sidewalk to minimize traffic accidents [35]. Individuals at risk of injury require education [36].

Different educational courses can be provided for different age/gender/occupational groups based on demographics and epidemiologic features, and legislation to compel the use of protection instruments and helmets in high-risk jobs and to penalize persons who break road laws is needed [37]. Finally, the importance of SCI physiotherapy and rehabilitation should be considered.

## Data Availability

The data generated in this study are available from the corresponding author on reasonable request.
